# Cytotoxic and antibacterial activities of endophytic fungi isolated from plants at the National Park, Pahang, Malaysia

**DOI:** 10.1186/1472-6882-9-46

**Published:** 2009-11-21

**Authors:** Nurul AMN Hazalin, Kalavathy Ramasamy, Siong Meng Lim, Ibtisam Abdul Wahab, Anthony LJ Cole, Abu Bakar Abdul Majeed

**Affiliations:** 1 Collaborative Drug Discovery Research (CDDR) Group, Faculty of Pharmacy, Universiti Teknologi MARA (UiTM), 40450 Shah Alam, Selangor Darul Ehsan, Malaysia; 2 Institute for the Study of Natural Remedies (iKUS), Faculty of Pharmacy, Universiti Teknologi MARA (UiTM), 40450 Shah Alam, Selangor Darul Ehsan, Malaysia; 3 School of Biological Sciences, University of Canterbury, Private Bag 4800, Christchurch, New Zealand; 4 Brain Research Laboratory, Faculty of Pharmacy, Universiti Teknologi MARA (UiTM), 40450 Shah Alam, Selangor Darul Ehsan, Malaysia

## Abstract

**Background:**

Endophytes, microorganisms which reside in plant tissues, have potential in producing novel metabolites for exploitation in medicine. Cytotoxic and antibacterial activities of a total of 300 endophytic fungi were investigated.

**Methods:**

Endophytic fungi were isolated from various parts of 43 plants from the National Park Pahang, Malaysia. Extracts from solid state culture were tested for cytotoxicity against a number of cancer cell lines using the MTT assay. Antibacterial activity was determined using the disc diffusion method.

**Results:**

A total of 300 endophytes were isolated from various parts of plants from the National Park, Pahang. 3.3% of extracts showed potent (IC_50 _< 0.01 μg/ml) cytotoxic activity against the murine leukemic P388 cell line and 1.7% against a human chronic myeloid leukemic cell line K562. *Sporothrix *sp. (KK29FL1) isolated from *Costus speciosus *showed strong cytotoxicity against colorectal carcinoma (HCT116) and human breast adenocarcinoma (MCF7) cell lines with IC_50 _values of 0.05 μg/ml and 0.02 μg/ml, respectively. Antibacterial activity was demonstrated for 8% of the extracts.

**Conclusion:**

Results indicate the potential for production of bioactive agents from endophytes of the tropical rainforest flora.

## Background

Endophytes are microbial entities that live within living tissues of plants without apparently any deleterious consequences [1]. Their biological diversity, especially in temperate and tropical rainforests, is large. Each plant species may be host to a number of endophytes [2].

Since the discovery of the world's first billion-dollar anticancer compound - paclitaxel (Taxol) - could be biosynthesized by *Pestalotiopsis microspora*, a fungus that colonizes the Himalayan yew tree, interest in studying such endophytes for their medicinal potential has grown tremendously [3]. To date, endophytes have been most extensively studied for their ability to produce antibacterial, antiviral, anticancer, antioxidants, antidiabetic and immunosuppressive compounds [1]. Their study is expected to become an important component in the production of new natural bioactive products.

Only a few studies on endophytic fungi from Malaysian plant species have been conducted so far. The current study was undertaken to investigate this biodiversity and to isolate and screen endophytic fungi with cytotoxic and antibacterial activities from medicinal plants collected from two locations in the National Park, Pahang, Malaysia.

## Methods

### Source of endophytic fungi

Plant materials were obtained from the National Park, Pahang, Malaysia in June, 2007. Two different locations, Kuala Keniam (KK) and Kuala Trenggan (KT), where medicinal plants could be found in abundance were selected for sampling. Chosen parts from individual plants were collected and stored at 4°C until used. All plant samples were identified by Kamaruddin Saleh of the Forest Research Institute of Malaysia (FRIM) and were deposited in the herbarium at the Faculty of Pharmacy, Universiti Teknologi MARA, Shah Alam, Malaysia.

### Isolation of endophytic fungi

Isolation of endophytes from the 43 plant samples was carried out as described by Strobel *et al*., [4] but with minor modifications. Plant samples, which included leaves, stems, roots, rhizomes, flowers, fruits and bark, were washed under running tap water for 10 min followed by immersion in 70% EtOH for 1 min and in NaOCl (2.5% - 5.25%) for 3 min, drained and immersed in 70% EtOH again for 30 sec. Finally, the samples were rinsed with sterile d.H_2_O. Each plant sample was cut aseptically into 1 cm long segments. The cut surfaces of the segments were placed on petri dishes containing potato dextrose agar (PDA) (Oxoid) supplemented with chlortetracycline HCL (50 μg/ml, Sigma) and streptomycin sulphate (250 μg/ml, Sigma) at 28°C. Pure cultures were then transferred to PDA plates free of antibiotics and maintained in the culture collection of the Collaborative Drug Discovery Research (CDDR) Group, UiTM, Malaysia. For investigations of biological activity, the endophytes were cultivated for 14 days on PDA plates at 28°C.

### Semipolar extraction of fungal cultures

Crude endophytic extracts were prepared as described by Lang *et al*., [5] but with slight modifications. Endophytic cultures (five plates per fungus) were homogenized and transferred to a 500 ml conical flask filled with 250 ml EtOAc (Merck) and left to stir overnight at room temperature. The mixture was filtered through Whatman No.1 filter paper, after which Na_2_SO_4 _(40 μg/ml, Merck) was added to further remove the aqueous layer within the mixture. The mixture was then transferred to a round bottom flask and dried using a rotary evaporator. The resultant extract was dissolved in 1 ml of dimethyl-sulfoxide (DMSO) (Sigma) and kept at 4°C as stock solution.

### Cytotoxic activity

Human chronic myeloid leukemic, K562 (ATCC CCL - 243), murine leukemic, P388 (ATCC TIB 63), human colorectal carcinoma, HCT116 (ATCC CCL - 247) and human breast adenocarcinoma, MCF7 (ATCC HTB - 22) cell lines were purchased from the American Type Culture Collection (ATCC), Manassas, VA, USA. All cell lines were cultured in RPMI 1640 (Sigma) supplemented with 10% heat inactivated fetal bovine serum (FBS) (PAA Laboratories) and 1% penicillin/streptomycin (PAA Laboratories). Cultures were maintained in a humidified incubator at 37°C in an atmosphere of 5% CO_2_.

Cytotoxicity of extracts at various concentrations (0.01 - 100 μg/ml) was assessed using the 3-(4, 5-dimethylthiazol-2-yl)-2, 5-diphenyl tetrazolium bromide (MTT) (Sigma) assay, as described by Mosmann, 1983 [6] but with minor modification, following 72 h of incubation. Assay plates were read using a spectrophotometer at 520 nm. Data generated were used to plot a dose-response curve of which the concentration of extract required to kill 50% of cell population (IC_50_) was determined. Cisplatin (Mayne Pharma) and tamoxifen (Dynapharm), which are both established chemotherapeutics, were used for comparison. Cytotoxic activity was expressed as the mean IC_50 _(± standard deviation) of three independent experiments.

### Antibacterial activity

The crude extracts of the 300 endophytic fungi were tested against *Bacillus subtilis *(ATCC 6633), *Micrococcus luteus *(ATCC 10240), *Staphylococcus aureus *(ATCC 25923), *Escherichia coli (*ATCC 25922) and *Pseudomonas aeruginosa *(ATCC 27853). Antibacterial activity was determined using the disc diffusion method according to the National Committee for Clinical Laboratory Standards (NCCLS) [7]. Pre-warmed Mueller-Hinton agar (MHA) (Oxoid) plates were seeded with 10^7 ^- 10^8 ^cfu suspension of test bacteria. Endophytic extracts (10 μl) dissolved in DMSO (1 mg/ml) were pipetted (10 μl) onto sterile paper discs (6 mm diameter, Oxoid) and placed onto the surface of inoculated agar plates. Gentamicin sulphate (10 μg, Oxoid) was used as the positive control. Plates were incubated at 37°C for 48 h. Antibacterial activity was expressed as the diameter of the inhibition zone (mm) produced by the extracts.

## Results and discussion

A total of 300 endophytes were isolated from 43 plants found at two different locations (Kuala Keniam and Kuala Trenggan) within the National Park, Pahang, Malaysia (Table 1). Of the total endophytes obtained, 70.0% were isolated from plants at Kuala Keniam, and the remaining from Kuala Trenggan. Relatively greater distribution of endophytes was found within leaf (48.7%), stem (25.7%) and root (16.3%) samples compared to other segments (9.3%, including flower, fruit, rhizome and bark) of the plants. *Ardisia colorata *(laloh, local name) was found to host the highest number of endophytes (14 isolates), followed by *Molineria latifolia *(13 isolates) and *Zingerberaceae *sp., KT43 (13 isolates).

**Table 1:** Endophytic fungi isolated from various parts of plants from the National Park, Pahang

Plant code*	Plant species	No. of isolates obtained from:					Total
			
		Leaf (L)	Stem (S)	Root (R)	Flower (FL)	Other	
KK1	*Donax grandis *Ridl.	2		2		1	5
KK2	*Angiopteris evecta *(G. Forst.) Hoffm.	2	2			1	5
KK3	*Clidemia hirta *(L.) D. Don	5	3	3			11
KK4	*Palmae sp.*	3	2				5
KK5	*Amischotolype mollisima *Hassk.	4	2	1	2		9
KK6	*Cnestis palala *(Lour.) Merr.	3	4	1			8
KK7	*Sindora coriacea *(Baker) Prain.	5				2	7
KK8	*Antiaris toxicaria *(Pers.) Lesch	6					6
KK9	*Phyllagathis rotundifolia *(Jack) Blume	3	6	3			12
KK10	*Catunaregam spinosa *(Thunb.) Tirveng	3	2			2	7
KK11	Unidentified		3				3
KK12	*Tacca integrifolia *Ker Gawl.	4	2	3			9
KK13	*Ixora grandiflora *Zoll. & Mor.	2				1	3
KK14	*Ampelocissus cinnamomea *Planch.	3	5	1			9
KK15	Unidentified					1	1
KK16	*Tetracera indica *Merr.	3		2			5
KK17	*Chroesthes longofolia *(Wight) B. Hansen	4		2			6
KK18	*Ancistrocladus tectorius *(Lour.) Merr.	3	2				5
KK19	*Ardisia colorata *Wall. Ex Roxb.	5	4	1		4	14
KK20	*Dendropanax laurifolius *(E. March.) Dcne. & Planch.ex R. C. Schneid.	2	2	1			5
KK21	*Zingerberaceae sp.*	2			2	1	5
KK22	*Clerodendrum deflexum *Wall.	3	5	1			9
KK23	*Cleistanthus sp.*	3	1				4
KK24	*Koompassia excelsa *(Becc.) Taub	5				2	7
KK26	*Anacolosa frutescens *(Blume) Blume	4	3				7
KK27	*Justicia sp.*	3		4			7
KK28	*Psychotria condensa *King & Gamble	3		2			5
KK29	*Costus speciosus *(J. Konig) Sm	3	2	2	3		10
KK30	*Zingerberaceae sp.*	3	1		2	2	8
KK31	*Brassaiopsis polyacantha *(Wallich) Banerjee	4		3			7
KK32	*Eurycoma longifolia *Jack	5		1			6
KT33	*Leptonychia caudata *(Wall. ex G.Don) Burrett	3	1	1			5
KT34	*Araceae sp.*	4	2	2			8
KT35	*Dioscorea hispida *Dennst.	2	3				5
KT36	*Phyllanthus pulcher *Wall. ex Müll. Arg.	3	1	1			5
KT37	*Mimosa sp.*	3	2	1			6
KT38	*Thottea sp.*	4	4	3			11
KT39	*Molineria latifolia *(Dryand. ex W.T.Aiton) Herb. ex Kurz	8	2	3			13
KT40	*Caesalpinia parviflora *Prain	3	3				6
KT41	*Strychnos ignatii *P. J. Bergius.	4		1			5
KT42	*Centotheca lappacea *(L.) Desv.	2		1			3
KT43	*Zingerberaceae sp.*	6	4	3			13
KT44	*Rotheca serrata *(L.) Steane & Mabb.	4	4		2		10

**Total**		**146**	**77**	**49**	**11**	**17**	**300**

*KK - Kuala Keniam; KT - Kuala Trenggan

Cytotoxicity of the extracts against P388 and K562 cell lines is shown in Table 2. Generally, the extracts were found to be more effective against P388 than the K562 cell line. Nearly half (47.6%) of the extracts showed activity (IC_50 _of < 10 μg/ml) against P388 compared with 25% active against K562. These values were within the cutoff point of the National Cancer Institute criteria for cytotoxicity (IC_50 _< 20 μg/ml) in the screening of crude plant extracts [8].

**Table 2:** Percentage of endophytes showing cytotoxic activity against P388 and K562 cell lines

IC_50 _(μg/mL)	Endophytes (%)	
	
	P388	K562
< 0.01	3.3	1.7
0.01-0.099	5	3
0.1-0.999	7	5
1-9.999	32.3	15.3
10-100	37.3	42
> 100	14.7	28.7
Not available*	0.3	4.3

*Not able to obtain IC_50 _after three independent tests.

At IC_50 _levels < 1 μg/ml, 15.3% of extracts were active against P388 and 9.7% against K562 cell line. Very potent cytotoxicity (defined as IC_50 _< 0.01 μg/ml) against P388 was shown by 3.3% of the extracts and 1.7% against K562. The ten endophytic extracts that showed very potent cytotoxic activity (IC_50 _< 0.01 μg/ml) against P388 showed greater cytotoxicity than the pure compounds paecilosetin (IC_50 _= 3.2 μg/ml) and farinosone (IC_50 _= 1.1 μg/ml) isolated from an enthomopathogenic fungi,*Paecilomyces farinosus *[9] and penicillenol (IC_50 _= 2.6 μg/ml) from *Penicillium *sp. GQ-7, an endophytic fungi [10]. When compared with reported activity of compounds from marine organisms, 46 of the extracts (IC_50 _< 1 μg/ml) showed greater potency than kulokekahilide-1, a cytotoxic depsipeptide from Chepalaspidean mollusk *Philinopsis speciosa *(IC_50 _= 2.1 μg/ml) when tested against P388 [11]. The five extracts with IC_50 _< 0.01 μg/ml against K562 were found to be more potent than the crude extract of *Aspergillus *sp. B-F-2 (IC_50 _= 50 μg/ml) when tested against the same cell line [12]. However, these extracts were found to be less cytotoxic than chaetominine, a cytotoxic alkaloid produced by an endophyte *Chaetomium *sp. IFB-E015 which had an IC_50 _of 0.008 μg/ml against K562 [13].

The extract of strain KK29FL1, a *Sporothrix *sp., that showed the greatest activity against P388 and K562 was further assayed against HCT116 and MCF7 cell lines and shown to exhibit strong cytotoxicity against HCT116 (IC_50 _= 0.05 μg/ml) and MCF7 (IC_50 _= 0.02 μg/ml). This extract showed higher cytotoxic activity than that reported for cisplatin (Mayne Pharma) against HCT116 (IC_50 _= 0.60 μg/ml) and tamoxifen (Dynapharm) against MCF7 (IC_50 _= 0.04 μg/ml). When tested against HCT116, strain KK29FL1 was found to be more cytotoxic than rubrofusarin B, IC_50 _= 4.5 μg/ml [14] and chaetominine, IC_50 _= 11.3 μg/ml [13] isolated from endophytes of *Cyndon dactylon *and *Adenophora axilliflora *respectively. Strain KK29FL1 was also found to be more cytotoxic than beauvericin and bikaverin (IC_50 _= 1.42 μg/ml and 0.161 μg/ml respectively) isolated from an endophytic *Fusarium oxysporum *[15], when tested against MCF7.

Only 24 isolates (8%) displayed antibacterial activity against at least one test microorganism with inhibition zones of 7 to 8 mm as shown in Table 3. Approximately half of the active isolates displayed inhibitory activity against *E. coli*, however, none of the isolates were as potent as gentamicin sulphate. In contrast, other studies have shown that endophytes are a good source of antibacterial agents. Guimaraes *et al*. [16] screened extracts from 39 endophytic fungi isolated from *Viguiera arenaria *and *Tithonia diversifolia*, resulting in 5.1% active extracts against *S. aureus *and 25.6% active extracts against *E. coli*. An extract of *Streptomyces *sp. (SUK 06) isolated from the stem of a Malaysian plant was found to be as effective as oxacillin against *B. subtilis *[17]. Kakadumycin from *Streptomyces *sp. NRRL 30566 isolated from *Grevillea pteridifolia *was effective against *S. aureus *[18]. Munumbicin B and D that was isolated from *Streptomyces *sp. NRRL 30562, an endophytic fungus of *Kennedia nigriscans*, possessed antibacterial activity as effective as vancomycin against *S. aureus *[19].

**Table 3:** Antibacterial activity of extracts

Endophytes	Antibacterial activity (mm)				
	
	Ec	Sa	Pa	Ml	Bs
KK1L2	7	-	-	-	-
KK3R3	7	-	-	-	-
KK4L1	7	-	-	-	-
KK5L1	7	-	-	-	-
KK5L4	7	-	-	-	-
KK5R1	7	-	-	-	-
KK8L3	7	-	-	-	-
KK9L2	7	-	-	-	-
KK9S3	-	7	-	-	-
KK9S5	7	-	-	-	-
KK9R1	7	-	-	-	-
KK11S2	-	-	7	-	-
KK12R2	7	-	7	-	-
KK16L1	-	8	7	-	-
KK16L3	-	7	-	-	-
KK18S1	7	-	-	-	-
KK19S3	7	-	-	-	-
KK27R1	-	-	7	-	-
KK30S1	-	-	-	7	-
KK30RH1	-	-	8	8	-
KT33L1	-	-	8	-	-
KT34L3	-	-	7	-	7
KT34S1	-	-	7	-	7
KT43L4	-	-	7	-	-

Gentamycin	25	11	15	30	25

KK - Kuala Keniam; KT - Kuala Trenggan; L - Leaf; S - Stem; R - Root; RH - Rhizome Test microorganisms: Ec - *Escherichia coli*; Sa - *Staphylococcus aureus*; Pa - *Pseudomonas aeruginosa*; Ml - *Micrococcus luteus*; Bs - *Bacillus subtilis*.

- : None detected.

## Conclusion

In conclusion, this preliminary screening of rainforest fungal endophytes revealed their potential to yield potent bioactive compounds for drug discovery programmes. Extract KK29FL1, a *Sporothrix *sp., showed very potent cytotoxic effect indicating its possible potential for development as an anti-cancer drug and warrants further investigation.

## Competing interests

The authors declare that they have no competing interests.

## Authors' contributions

KR was the principal investigator who participated in the designing of the study, plant collection and writing of the manuscript. NAMNH participated in the plant collection, overall conduction of experiments and writing the manuscript. LSM and ALJC participated in the planning of the study, plant collection and writing the manuscript. IAW and ABAM participated in the planning of the study.

## Pre-publication history

The pre-publication history for this paper can be accessed here:

http://www.biomedcentral.com/1472-6882/9/46/prepub
